# The Complexity of Neuroenhancement and the Adoption of a Social Cognitive Perspective

**DOI:** 10.3389/fpsyg.2015.01880

**Published:** 2015-12-01

**Authors:** Arnaldo Zelli, Fabio Lucidi, Luca Mallia

**Affiliations:** ^1^Department of Movement, Human and Health Sciences, University of Rome “Foro Italico”, Rome, Italy; ^2^Department of Psychology of Development and Socialization Processes, Sapienza University of Rome, Rome, Italy

**Keywords:** neuroenhancement, students, social cognitive models, doping, substance use

## Abstract

This contribution attempts to provide a broad perspective to the psychological study of neuroenhancement (NE). It departs from the assumption that, as the use of performance enhancing substances in sport, the use of substances with the aim of improving one’s cognitive, motivational and affective functioning in academic domains is a goal-directed behavior. As such, its scientific study may very well benefit from an analysis taking into account the psychological processes regulating people’s behavioral intentions and decisions. Within this broad framework, this contribution addresses several issues that currently seem to characterize the debate in the literature on neuroenhancement substances (NES) use. The first conceptual issue seeks to determine and define the “boundaries” of the phenomenon. The second issue concerns the empirical evidence on the prevalence of using certain substances for the purpose of NE. Finally, there is a debate around the ethical and moral implications of NE. Along these lines, the existing psychological research on NE has adopted mainly sociological and economic decision-making perspectives, greatly contributing to the psychological discourse about the phenomenon of NE. However, we argue that the existing psychological literature does not offer a common, explicit and integrated theoretical framework. Borrowing from the framework of doping research, we recommend the adoption of a social cognitive model for pursuing a systematic analysis of the psychological processes that dynamically regulate students’ use of NES over time.

## Premises

The use of pharmacological substances to enhance performance is an issue psychologists have thoroughly investigated in the sport context. In this context, a broad psychological perspective focusing on the social-cognitive processes regulating one’s intentions and use of performance enhancement substances has been largely adopted by many scholars in recent years (see Ntoumanis et al., 2014, for a review). Similarly, the use of substances with the aim of improving one’s cognitive, motivational and affective functioning in academic and work contexts has also recently emerged and been debated as a critical research issue in the literature on neuroenhancement (NE) and cognitive enhancement (e.g., Zohny, 2015).

We believe this debate is currently focusing on three clearly distinct—and yet intimately related—issues. There is a conceptual focus which seeks to determine and define the “boundaries” of the phenomenon. Some boundaries stress the distinction between pharmacological and non-pharmacological substances to enhance performance. Other boundaries instead refer to the distinction between “hot” (e.g., mood, motivation) and “cold” (e.g., attention, memory) cognitions, and to the general notion that cognitive enhancement seemingly only matters for the latter type of cognitions (Zohny, 2015). Finally, there are boundaries stressing the contexts in which it is plausible or relevant to discuss NE (e.g., work places, educational settings) and those to which the term of NE instead does not apply (e.g., recreational settings, sport settings). There also is an empirical debate seeking to clarify the prevalence and social relevance of using certain substances for the purpose of NE (e.g., Maier and Schaub, 2015). There is also a debate around the ethical and moral implications of NE, with the literature primarily addressing issues ranging from personal safety, to the social responsibility of institutions, agencies or firms promoting or contributing to NE, to issues about a person’s character and his or her right to seek a good life (Schermer, 2008). This contribution briefly summarizes the key elements of these debates and, while recognizing the undisputable value these debates have for scientific progress, also argues that they are undermined by a lack of explicit reference to a clear theoretically-grounded psychological perspective. We believe that the adoption of a theoretical psychological perspective, as in the case of existing doping research, would favor a shift from insightful and yet seemingly endless debates to prospective research and intervention programs that could clarify and possibly resolve some of these debates. In the remaining sections of this paper, we attempt to sustain and justify this core belief.

Finally, it is important to note that the present contribution unfolds with an exclusive focus on academic or educational contexts. Typically, these contexts offer clear-cut and broadly acknowledged behavioral criteria and protocols for referring to and observing individuals’ performances. Furthermore, as also suggested by Kipke (2013), academic examinations and testing might warrant special attention, as performance outcomes clearly rely on one’s cognitive functioning and capacities. Third, NE in these settings also raises issues regarding the integrity and validity of academic examinations and testing results. As a concluding note, academic and educational settings are also the contexts which have often been the target of empirical studies on NE (e.g., Smith and Farah, 2011; Franke et al., 2014).

## Definitional and Conceptual Issues Concerning Neuroenhancement

Should NE be considered a complex property of some substances currently is a matter of debate, and the scientific evidence and general understanding of this proposition seems far from having been ascertained or confirmed (Zohny, 2015). Even if one departed from the definition of NE that in recent years has been shared by scholars and referred to as “*.… the misuse of prescription drugs*, *other illicit drugs, or alcohol for the purpose of enhancing cognition, mood, or prosocial behavior in academic or work related contexts*” (e.g., Maier and Schaub, 2015, p. 156), we feel that this definition, despite being extremely clear, still needs further consideration or clarification.

First, some NE studies distinguish among prescribed-substances (e.g., Methylphenidate, Modafinil, Amphetamines, etc), substances of abuse (e.g., Alcohol, Cannabis, Cocaine) and over-the-counter substances or drugs (e.g., caffeinated products and food supplements), the so called “soft enhancers” or “life style” drugs (e.g., Franke et al., 2014; Maier and Schaub, 2015). Second, clarification seems warranted when one considers the extent to which NE must or needs to be conceived with respect to behavioral rather than cognitive performance criteria (e.g., a substance enhances one’s memory which, in turn, affects and positively contributes to one’s exam grades). With this in mind, some scholars (e.g., Zohny, 2015) distinguish substances’ effects on mood or motivational processes from their effects on other processes, such as attention or memory, and go on in suggesting that the latter type of effects specifically constitutes cognitive enhancement. Whether “cognitive” only refers to what is traditionally seen as “cold” cognition or, rather, whether motivational and emotional processes legitimately represent parts of one’s cognitions, is an issue that has been long debated in classical work (e.g., Pessoa, 2008).

In the context of the present contribution, it seems important to us to highlight another issue that perhaps has relevant assonances with the distinction between “cold” and “hot” cognitions. One’s use of cognitive enhancement substances legitimately may call upon two broadly alternative cases. The first envisions the possibility that one may use a given substance to improve his or her “*effort*” as a means of performance (e.g., Ritalin to stay awake and study for a longer time). The second envisions the possibility that one may use a given substance to improve specific cognitive functions or tasks (e.g., memory recall or problem solving). Both cases highlight a critical issue in any psychological analysis, that of one’s goals for choosing a particular course of action. At any rate, to what extent any or both of these cases must be considered “cognitive enhancement” has not yet been addressed by the existing literature. At a minimum, however, it seems plausible to hypothesize that users of cognitive enhancement substances might primarily be interested in achieving their best (academic) performance outcomes, rather than in the processes underlying any particular outcome.

We think the above issues, despite their peculiarities, offer some ground for consensus. The use of neuroenhancement substances (NES), by students or professionals, reflects a person’s conscious or deliberate intentions, at least in the case of unsupervised use of psychoactive substances by healthy individuals. Furthermore, no matter what the NES chemical and medical properties are with respect to the enhancement of specific cognitive capacities (e.g., memory), we think that there is consensus in the literature on the general view that individuals pursue enhancement goals with the intention of influencing actual behavioral performance.

## The Prevalence of Neuroenhancement Substance Use

A large number of recent empirical NE studies have estimated the prevalence of NES use. However, it seems difficult to draw a precise and reliable map of its diffusion, as prevalence estimates often vary widely depending upon sampling criteria, measurements, and demographic or contextual factors. For instance, Smith and Farah (2011), in reviewing 28 epidemiological studies on the prevalence of non-medical prescription drug use in American and Canadian students, reported a lifetime use of stimulants for non-medical purposes ranging from 5.3 to 55%. More recently, Franke et al. (2014) have reviewed studies reporting prevalence rates for NES use that range from 1 to 20%.

The issue of reliably assessing prevalence rates has also characterized doping research, and the distinction between legal and illegal substances has definitely contributed to establishing valid estimates of doping use in sport settings (Mallia et al., 2013). In a similar fashion, it is plausible that the distinction among non-medical prescription drugs, drugs of abuse, and soft-enhancers (e.g., caffeine) in the NE literature might contribute to a correct assessment of prevalence estimates.

Generally speaking, however, the estimation of prevalence of NES use, as for performance enhancing substances (PES) use in sport, remains a complex process and many methodological issues could influence it and lead to increasing variability and differences in findings across studies. For instance, while social desirability biases might easily come into play in the assessment of doping substance use in the face of explicit sport law regulations against their adoption, the lack of any clear-cut social or legal norms about NES may pose complex challenges for correct or agreed-upon prevalence rates.

## Ethical and Moral Issues Concerning Neuroenhancement

There is an important debate concerning the ethical issue related to the use of NES. Some scholars argue that, especially in the context of examinations, this behavior might be considered cheating, because its use may alter performance (Schermer, 2008), as in the well-known case of doping in sports. There are a number of parallels between the misuse of NES in academic settings and doping in sport. In both contexts, an individual is misusing a substance that has legitimate medical value with the purpose of increasing one’s own performance. As in the field of doping research (see, for istance, Petroczi, 2013), several scholars have debated the ethical and moral implications of using NES in academic or educational settings (e.g., Kipke, 2013; Zohny, 2015).

At the same time, there are also some clear differences between the use of NES and the use of doping substances. In sport contexts, there is a clear and well-accepted distinction between which substances and protocols are illicit (illegal performance enhancing substances) and which are not (legal performance enhancing substances). In educational and academic contexts, at least until recently, law or binding regulations concerning the use of cognitive enhancing substances were lacking. Some universities, in fact, have recently clarified in their own academic conduct policies that the use of prescription medications aimed at enhancing academic performance falls in the category of “academic dishonesty” (e.g., Duke University: Policy on academic dishonesty; URL: https://studentaffairs.duke.edu/conduct/z-policies/academic-dishonesty), even though policies of this sort are still a matter of debate (e.g., Schermer, 2008; Dubljević, 2013). Interestingly, Dodge et al. (2012) have separately assessed how individuals judge others who use performance enhancing drugs both in athletic and academic domains. Not surprisingly, their findings suggest that people tend to consider the use of NES to enhance academic performance as more acceptable than doping substance use in sport.

One could reasonably argue that the lack of clear-cut norms and regulations for the use of NES makes the latter unfit for being treated as a case of cheating. Nonetheless, there are some actions or behaviors that, despite not being clear violations of explicit rules or norms, allow one to gain some advantages over others and, as such, might be considered unfair. In the sport context, these behaviors fall under the rubric of “gamesmanship” (e.g., Lee et al., 2007). According to Vallerand et al. (1996), in order to approach the ethical evaluations of a given behavior, one needs to recognize the social origins of these evaluations, that is, the notion that they emerge over time by consensus within a social context. How individuals perceive the misuse of substances has important implications for prevention efforts. Thus, the use of NES might be evaluated positively when the emphasis and judgment criteria focus on one’s effort to perform well, and negatively when the emphasis and judgment criteria focus on one’s attempt to increase one’s own academic performance through the help of pharmacological aids, thus altering the integrity and validity of (his or her) academic examinations and testing results.

Faulmuller et al. (2013) emphasize that the indirect psychological costs of the use of NES is related to the ways people attribute performance to agents. Given that people tend to exaggerate the efficacy of cognitive enhancers, they might perceive NES users’ performance as not fully attributable to them. At any rate, individuals contemplating the use of NES may very well dwell upon the moral implications of using these substances and utilize their personal self-sanctions as internal deterrents. These possibilities imply and presuppose a strong link between NES use and moral reasoning, and this link is consistent with a well-grounded psychological literature addressing the relations between moral reasoning and the use of performance enhancing substances in sport-related contexts (e.g., Lucidi et al., 2008, 2013; Zelli et al., 2010).

## A Social Cognitive Perspective on Neuroenhancement

### The Theoretical Framework: Its General Principles and Hypotheses

From the previous sections of this contribution, it appears clear to us that the use of NES falls under the rubric of a goal-directed behavior and, as such, its scientific study may very well benefit from a psychological analysis presuming that NES use depends on self-regulation and on the mental processes intervening in behavioral intentions and decisions bounded to specific social contexts or situations. So stated, our view endorses key tenets of a social cognitive perspective on NES use, insofar the latter “*…. entails not only behavioral skill in self-managing environmental contingencies, but also the knowledge and the sense of personal agency to enact this skill in relevant contexts. Self-regulation refers to self-generated thoughts, feelings, and actions that are planned and cyclically adapted to the attainment of personal goals….*” (Zimmerman, 2000, pp. 13–14).

These general notions seem to be shared at least in part by psychological research that has adopted sociological and economic decision-making perspectives (see Sattler et al., 2014, for a thorough review). Much of this research (e.g., Müller and Schumann, 2011; Sattler and Wiegel, 2013; Wolff and Brand, 2013) broadly argues that the use of (or willingness to use) cognitive enhancement substances reflects an instrumental decision individuals make on the basis of the degree to which substance use “fits” their personal preferences, perceived opportunities and constraints. Consistent with this general hypothesis, empirical studies have focused on several classes of variables, ranging from considerations about the risks and benefits of particular cognitive enhancement drugs’ characteristics (e.g., Castaldi et al., 2012), to forms of social environmental effects (e.g., forms of social control, social pressure from significant others) influencing decisions about the use of enhancement substances (e.g., Glannon, 2008; Bavarian et al., 2013) to, finally, personal characteristics (e.g., cognitive test anxiety, lack of academic competencies) that may make individuals more vulnerable at the time of deciding whether to use cognitive enhancement substances (e.g., Tice and Baumeister, 1997; Klassen et al., 2008; Weyandt et al., 2009).

The focus on instrumental decisions also seems to characterize other NE research stressing the need for psychological theorizing (e.g., Wolff and Brand, 2013; Wolff et al., 2014). This research hypothesizes that NE is “*…the medically unsupervised use of presumably psychoactive substances by healthy individuals who expect this substance to be a functional means of enhancing their cognitive capacity…*” (Wolff et al., 2014, p. 2). This research very recently has moved on and utilized principles and constructs borrowed from occupational theories (e.g., demands, strain, burnout) to address the “means-to-end” NE hypothesis in educational settings (Wolff et al., 2014). This research has shown that the use of lifestyle drugs and prescribed NE drugs is more likely among university students who experience burnout, and that the use of NES worsens students’ psychological experience of academic demands and interferes with their motivational resources. These existing contributions have greatly contributed to the psychological discourse about NE.

This notwithstanding, it seems difficult to identify in this literature a common and explicit theoretical framework. On the contrary, and interestingly, doping-related psychological research has in recent years been able to adopt a broad social cognitive view that clearly and systematically put the study of performance enhancement substances on a qualitatively different level of theoretical analysis. According to this social cognitive view, doping substance use is a goal-directed behavior that is the expression of one’s intentional processes, and these intentions reflect the influence of socially construed belief systems. Illustratively, this broad view has found clear and distinct expressions in research that variously adopted either a “theory of planned behavior” approach (e.g., Lucidi et al., 2004; Lazuras et al., 2010; Mallia et al., 2013), a motivational orientation approach (e.g., Barkoukis et al., 2013) or an explicit social-cognitive integrative approach (e.g., Lucidi et al., 2008, 2013; Zelli et al., 2010; Lazuras et al., 2015). All these cases typically refer to belief structures, and these beliefs may specifically refer to either outcome beliefs guiding one’s behavioral attitudes about doping use, behavioral control beliefs concerning the means for reaching one’s own goals, personal and self-regulatory efficacy beliefs, or moral disengagement beliefs that one may adopt to counteract personal self-sanctions against doping use (e.g., Lucidi et al., 2008, 2013; Lazuras, 2015).

This belief-based social cognitive doping research has more recently been integrated by an additional social cognitive component, namely, one’s self-relevance appraisals of interpersonal and social situations eliciting doping use (Zelli et al., 2010, 2015). Theoretically, over time, this component would interact with belief systems in increasing the probability that people would show doping intentions and actual doping use.

We argue that the theoretical and empirical advances of doping research stand as a mature and plausible model for moving forward on NE research. In the following section, we describe, albeit in broad terms, some key elements of a possible social cognitive research program for the study of NE use.

### A Social Cognitive Research Program for Neuroenhancement

As an initial note, we believe that a social-cognitive model of NES use might nicely integrate some of the theoretical propositions that seem to have variously characterized recent NES studies. One proposition calls upon an incremental-functional view of NES use, and the hypothesis that students might be motivated and involved in performance enhancing practices that, over time, increasingly acquire high instrumental value (e.g., Sattler and Wiegel, 2013; Wolff and Brand, 2013). Another proposition calls upon belief systems which may build upon a link between one’s performance enhancement goals and the functional or moral implications of NES use as a purposive, goal-driven behavior.

We also believe that, at least in educational settings, research attention to constructs such as (a) students’ attitudes about NES, (b) prospective intentions toward NES use, (c) efficacy and self-regulatory beliefs about one’s own capacity to counteract social and internal pressures to use NES, (d) personal standards and justifications in favor or against NES use, and (e) students’ appraisals of the self-relevance of interpersonal situations eliciting NES use would have high scientific value. It would acknowledge and be consistent with the above theoretical propositions, as these social cognitive constructs recognize and encompass the dynamic and functional properties of one’s life and behavioral experiences with NES that existing literature has highlighted. More importantly, it would provide a single, unified, framework for theory development and assessment, allowing scholars to pursue a systematic analysis of the psychological processes that dynamically regulate students’ use of NES over time.

In our view, such a novel research focus should rely on and pursue some key research objectives. The first is concerned with the possibility of clearly establishing the empirical relations between people’s behavioral intentions and actual NES use. This first objective necessarily calls upon a second objective, namely, the adoption of longitudinal research designs allowing scholars to establish how behavioral intentions contribute to *changes* in NES use over time (i.e., controlling for behavioral stability). The third objective is concerned with the possibility of identifying the set of key social-cognitive variables regulating people’s NES behavioral intentions. As these variables operate in a system of dynamic relations, the empirical focus cannot merely address their unique contribution to behavioral NES intentions. Rather, it also needs to address how changes in the model of effects on behavioral intentions correspond to changes in the interrelations among key social cognitive variables and in their unique contributions. Consistent with a social-cognitive view of NES, the hypothesis of a system of interrelated variables influencing one’s behavioral intentions also calls upon the empirical possibility that this system is dynamically linked to the meaning people assign to relevant social and interpersonal situations possibly soliciting NES use. We believe this is a fourth critical objective for NES research, insofar as one’s intention to use NES might be strengthened or, alternatively, weakened by the degree to which social and interpersonal situations acquire personal relevance.

As a concluding note, we firmly believe that the social cognitive research perspective that has been briefly outlined above can provide, whatever its findings might be, the specific contours for any educational program that is interested in effectively addressing NES use and its implications in people’s daily lives and experiences.

## Author Contributions

AZ, FL, and LM substantially have equally contributed to the development and preparation of the manuscript. Furthermore, all authors have approved the final version of the manuscript. Finally, the authors have agreed to be accountable for all aspects of the manuscript in ensuring that questions related to the accuracy or integrity of any part of it are appropriately investigated and resolved.

### Conflict of Interest Statement

The authors declare that the research was conducted in the absence of any commercial or financial relationships that could be construed as a potential conflict of interest.
